# Rapid Identification of Adulteration in Edible Vegetable Oils Based on Low-Field Nuclear Magnetic Resonance Relaxation Fingerprints

**DOI:** 10.3390/foods10123068

**Published:** 2021-12-09

**Authors:** Zhi-Ming Huang, Jia-Xiang Xin, Shan-Shan Sun, Yi Li, Da-Xiu Wei, Jing Zhu, Xue-Lu Wang, Jiachen Wang, Ye-Feng Yao

**Affiliations:** 1 Shanghai Key Laboratory of Magnetic Resonance, College of Physics and Electronic Science, East China Normal University, Shanghai 200062, China; 15800959127@163.com (Z.-M.H.); 18366102713@163.com (J.-X.X.); riki941112@gmail.com (Y.L.); dxwei@phy.ecnu.edu.cn (D.-X.W.); jzhu@phy.ecnu.edu.cn (J.Z.); xlwang@phy.ecnu.edu.cn (X.-L.W.); jcwang@phy.ecnu.edu.cn (J.W.); 2 National Institutes for Food and Drug Control, Dongcheng District, Beijing 100050, China; shanshans112@163.com

**Keywords:** adulteration, oil identification, edible vegetable oils, relaxation fingerprints, low-field nuclear magnetic resonance spectroscopy

## Abstract

Most current approaches applied for the essential identification of adulteration in edible vegetable oils are of limited practical benefit because they require long analysis times, professional training, and costly instrumentation. The present work addresses this issue by developing a novel simple, accurate, and rapid identification approach based on the magnetic resonance relaxation fingerprints obtained from low-field nuclear magnetic resonance spectroscopy measurements of edible vegetable oils. The relaxation fingerprints obtained for six types of edible vegetable oil, including flaxseed oil, olive oil, soybean oil, corn oil, peanut oil, and sunflower oil, are demonstrated to have sufficiently unique characteristics to enable the identification of the individual types of oil in a sample. By using principal component analysis, three characteristic regions in the fingerprints were screened out to create a novel three-dimensional characteristic coordination system for oil discrimination and adulteration identification. Univariate analysis and partial least squares regression were used to successfully quantify the oil adulteration in adulterated binary oil samples, indicating the great potential of the present approach on both identification and quantification of edible oil adulteration.

## 1. Introduction

Edible vegetable oils are basic foodstuffs that are very commonly used worldwide. Many different types of commercial vegetable oils are currently available, which range from traditional and generally inexpensive canola oil, peanut oil, soy oil, and sunflower oil to more exotic and expensive flaxseed oil and olive oil. Although most commercially available edible vegetable oils are produced by responsible vendors, economic motivations have led to the unreported adulteration of vegetable oils with less expensive vegetable oil substitutes by unscrupulous vendors [1,2]. This is a serious issue not only because it represents consumer deception, but also because it can lead to health and safety issues [3]. Therefore, the identification of adulteration in edible vegetable oils represents an essential operation that must be conducted to protect consumers from unreported adulteration.

A number of conventional analytical techniques have been applied for identifying adulteration in edible vegetable oils, which include gas chromatography (GC) [4], high-performance liquid chromatography (HPLC) [5], and GC combined with mass spectrometry (GC-MS) [6]. However, these techniques are destructive, and often require laborious and time-consuming sample pretreatment and preparation. These issues have been addressed by the application of non-invasive analytical techniques requiring very little sample pretreatment and preparation, such as near-infrared (NIR) spectroscopy [7], mid-infrared (MIR) spectroscopy [8], Raman spectroscopy [9], fluorescence spectroscopy [10], electronic nose (e-nose) systems [11], ion mobility spectrometry (IMS) fingerprints [12], ultraviolet–visible (UV–Vis) spectroscopy [13], and high-resolution nuclear magnetic resonance (NMR) spectroscopy [14,15,16]. However, most of these approaches have limited practicality owing to the need for long analysis times, professional training, and high-cost instrumentation. Therefore, the development of relatively simple, accurate, and rapid approaches for identifying adulteration in edible vegetable oils is urgently required for supporting both public safety and business interests worldwide.

These issues can be potentially addressed by low-field NMR (LF-NMR) spectroscopy, which has been widely used as a powerful analytical tool in food analysis for quality monitoring and process supervision [17,18,19,20]. For example, Ancora et al. [21] demonstrated that LF-NMR relaxation measurements could be applied to determine the occurrence of adulteration in EVOO samples when mixed with four different edible vegetable oils. Moreover, Wang et al. [22] applied this methodology to detect the adulteration of sesame oil samples with soybean oil, and the adulteration ratios were estimated using principal component analysis (PCA) and partial least squares (PLS). Similarly, Zhu et al. [23] applied discriminant analysis (DA) for estimating the adulteration ratios of peanut oil adulterated with soybean oil, rapeseed oil, or palm oil based on LF-NMR relaxometry measurements. The accuracy of edible oil adulteration detection and analysis based on these LF-NMR relaxometry techniques has been further enhanced by applying machine learning algorithms, such as support vector machine (SVM) [24], convolutional neural network (CNN) [25], and combined SVM and CNN machine learning approaches [26]. These studies have demonstrated the substantial potential for applying LF-NMR relaxometry for the rapid identification of oil adulteration. However, subsequent research has demonstrated that the identification capability of conventional LF-NMR relaxometry is often limited by the fact that the relaxation properties of individual edible oils can be very similar to those of complex mixtures including two or more edible oils [27]. As a result, the relaxation properties of edible oils captured by conventional LF-NMR spectroscopy may lack the discrimination capability required for meeting the needs of public safety and business interests.

The present study addresses this issue by increasing the discrimination capability of LF-NMR relaxometry measurements for detecting and analyzing edible oil adulteration through the application of magnetic resonance fingerprinting (MRF) [28,29,30]. The relaxation fingerprints obtained for six types of edible vegetable oil, including flaxseed oil (FO), olive oil (OL), soybean oil (SO), corn oil (CO), peanut oil (PO), and sunflower oil (SFO), are demonstrated by PCA to have sufficiently unique characteristics to enable unambiguous identification. The application of PCA to the relaxation fingerprints enables the development of a novel three-dimensional (3D) coordination system for obtaining a unique identifier for any arbitrary vegetable oil sample. This identifier provides a direct and simple means of distinguishing between different edible vegetable oil samples when correlated with clusters associated with each specific type of vegetable oil within that characteristic coordination system. The proposed methodology is employed to investigate the adulteration of FO samples by various concentrations of SO in detail. Moreover, the application of univariate regression and PLS regression (PLSR) models is demonstrated to enable the SO concentration in adulterated FO oil samples to be determined easily, accurately, and quantitatively.

## 2. Materials and Methods

### 2.1. Oil Sample Preparation

A total of 28 edible vegetable oils were selected for this study, including 4 brands of SO, 4 brands of PO, 5 brands of CO, 5 brands of OL, 5 brands of FO, and 5 brands of SFO. All of the oil samples were purchased from a local market and were subjected to testing within the warranty period.

The adulteration of FO samples was investigated by adding SO in various concentrations of 10%, 20%, 30%, 50%, 70%, and 90% by volume using the brands randomly selected. Each mixture sample was prepared in a vial which was shaken for 30 s to ensure well-mixing. Another series of mixed FO-SO samples (20%, 30%, 40%, 50%, 70%, and 80%) were prepared using the same brands of SO and FO used as a validation set.

All oil samples were sealed in a glass tube and kept in a refrigerator at 4 °C prior to conducting NMR spectroscopy experiments.

### 2.2. NMR Measurements

All NMR measurements were conducted at a temperature of 28 °C using a VTMR20-010V-I NMR spectrometer (Niumag Corp., Ltd., Shanghai, China) with a magnetic field of 0.5 T, which corresponds to a ^1^H resonance frequency of 21.3 MHz.

### 2.3. NMR Relaxation Fingerprints

**Figure 1:**
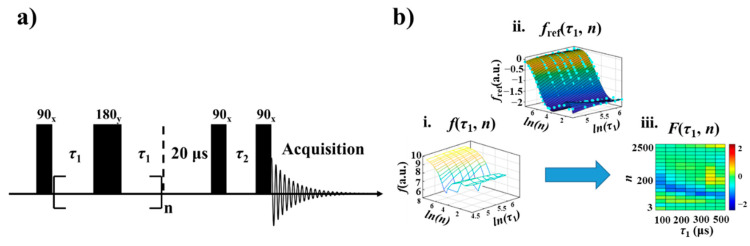
(**a**) The NMR pulse sequence used for recording relaxation fingerprints. (**b**) The procedure for generating relaxation fingerprints: (i). NMR relaxation data of a specific vegetable oil sample, which represents the signal intensity *f*(*τ*_1_, *n*) acquired using the pulse sequence in (**a**); (ii). reference relaxation data *f*_ref_(*τ*_1_, *n*), which is a curved surface obtained by fitting the average relaxation data of six different vegetable oil samples; (iii). heat map plot of the relaxation fingerprints *F*(*τ*_1_, *n*) = *f*(*τ*_1_, *n*) − *f*_ref_(*τ*_1_, *n*).

The developed NMR pulse sequence employed for the present study is illustrated in Figure 1a. The first part of this pulse sequence is a Carr-Purcell-Meiboom-Gill (CPMG) [31,32] train used to record the T_2_ relaxation behavior of the sample. The signs of x and y represent the phase of pulses (i.e., the 90° pulses and the 180° pulses), respectively. The delay *τ*_1_ and the number of loops *n* can be varied to select the different T_2_ relaxation components. Following the CPMG train is a combination of a 90° pulse and a delay *τ*_2_. Varying *τ*_2_ can introduce the T_1_ relaxation modulation into the NMR signals.

We first examined the relaxation behaviors of the six vegetable oil samples by applying the inversion recovery sequence and the above-defined CPMG sequence to obtain the T_1_ and T_2_ relaxation times of the samples, respectively [33,34]. The other experimental parameters applied were 4 scans, 90° pulse width of 2.72 μs, 180° pulse width of 5.12 μs, 50 ms acquisition time, and a 2 s recycle delay time to allow all the protons to return to thermal equilibrium. The T_1_ and T_2_ relaxation curves obtained for the six different vegetable oil samples are presented in Supporting Information (Figure S1), and the corresponding T_1_ and T_2_ values are listed in Table S1. The high degree of similarity in the relaxation behaviors of the oil samples clearly demonstrates the need for applying more distinguishable NMR relaxation fingerprints in the identification process.

The NMR relaxation fingerprints are two-dimensional (2D) characteristic NMR relaxation data that can be recorded by varying the values of *τ*_1_ (the 1st dimension) and *n* (the 2nd dimension) in a stepwise fashion. Accordingly, the relaxation fingerprints experiments employed *τ*_1_ values of 100 μs, 150 μs, 200 μs, 250 μs, 300 μs, 400 μs, and 500 μs, and *n* values of 3, 5, 10, 20, 40, 80, 100, 150, 200, 250, 300, 400, 500, 700, 900, 1200, 1500, and 2500. Meanwhile, the value of *τ*_2_ was fixed at 100 ms in all experiments. Based on the above experimental conditions, recording one NMR relaxation fingerprint dataset takes about 15 min in total for one sample.

The procedure employed for generating the NMR relaxation fingerprints is illustrated in Figure 1b. Applying the pulse sequence in Figure 1a with the above-defined parameters for a specific oil sample produces a series of NMR free decay signals of intensity *f* under the stepwise variation in *τ*_1_ and *n*. The signals were normalized by dividing the intensity of the signal from the single pulse excitation experiment. The resulting 2D relaxation data *f*(*τ*_1_, *n*) is plotted in Figure 1b(i). The same process is applied to six different vegetable oil samples, and the average intensity of the 2D relaxation data for the six samples is plotted as the reference signal *f*_ref_(*τ*_1_, *n*) in Figure 1b(ii). Finally, the NMR relaxation fingerprint of the specific oil sample is obtained as *F*(*τ*_1_, *n*) = *f*(*τ*_1_, *n*) − *f*_ref_(*τ*_1_, *n*), which is plotted in the form of a heat map in Figure 1b(iii). All the data processing procedures were performed using Matlab software (Matlab R2020b, Mathworks, Natick, MA, USA).

### 2.4. Statistical Analysis

The original relaxation fingerprint data were normalized by dividing the intensity of the signal acquired by using the single pulse excitation experiment with the same experimental conditions. Edible oil analysis methods have relied heavily on PCA owing to its high reliability and versatility [35,36,37]. In this work, PCA was applied to verify the capability of the relaxation fingerprints approach in edible vegetable oils discrimination. In PCA, the intensity values of the eight regions, named from A to H (Figure 3a), from the relaxation fingerprints of the 28 samples form a 28 × 8-matrix as the input data. The data were scaled by mean centering (Ctr). The matrix-type to analyze for PCA is a correlation matrix, and the singular values decomposition (SVD) algorithm was used for matrix decomposition. The loadings, which are the coefficients for principal components, are obtained by calculating the eigenvector of the correlation matrix.

The univariate linear regression analysis and PLSR of relaxation fingerprints data were performed to quantitatively detect the flaxseed oil adulterated with soybean oil. 3 SO samples, 4 FO samples, and 6 mixed FO-SO samples were randomly assigned to the calibration dataset. The remaining 1 SO, 1 FO, and 6 mixed FO-SO samples were used as the validation dataset. Univariate linear regression analysis used the intensity of B, E, G region in the relaxation fingerprint as independent variables, and used the true adulteration ratio as a dependent variable. In PLSR analysis, the intensity values of the eight regions from the relaxation fingerprints of the samples in the calibration set and validation set were considered as independent variables, and the adulteration ratios of the samples were considered as a dependent variable. Accordingly, two matrices with the dimension of 13 × 9 and 8 × 9 were formed and analyzed. SVD was used to compute extracted factors, and the cross-validation uses the leave-one-out scheme, leaving out one observation each time and using other observations to construct the model and predict responses for the observation [38]. Both univariate and multivariate statistical analyses were performed with the software OriginPro 2021 (OriginLab Corporation, Northampton, UK) and *p* < 0.05 was considered to be statistically significant.

## 3. Results and Discussion

### 3.1. Relaxation Fingerprints

**Figure 2:**
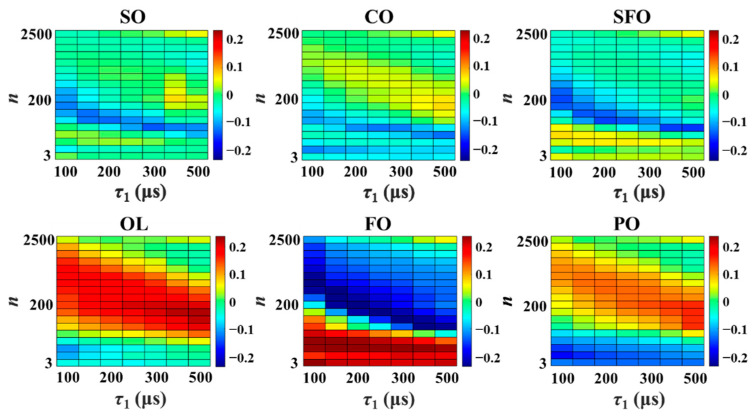
The NMR relaxation fingerprints in the form of heat map plots for six edible vegetable oils, including soybean oil (SO), corn oil (CO), sunflower oil (SFO), olive oil (OL), flaxseed oil (FO), and peanut oil (PO). Based on the experimental parameters described in the experimental section, recording one NMR relaxation fingerprint dataset takes about 15 min totally.

The NMR relaxation fingerprints obtained in the form of heat map plots for single brands of the six vegetable oils are presented in Figure 2. The NMR relaxation fingerprints obtained for a number of other oil brands are presented in Supporting Information (Figures S2–S4). It was observed that while the T_1_ and T_2_ relaxation behaviors of the oil samples are highly similar, the NMR relaxation fingerprints of the six edible vegetable oils differ widely, and clearly represent an objective basis for oil identification. We note that, from a chemical point of view, edible vegetable oils are mainly composed of triglycerides linked to various saturated fatty acids, polyunsaturated, and monounsaturated fatty acids [39]. However, due to the different local molecular arrangements, the environments of the oil molecule in different oils often differ, resulting in the different local molecular dynamics and, in turn, the different relaxation properties, including the T_1_ and T_2_ relaxation times [40]. In addition, trace paramagnetic metal ions (Iron, Manganese, etc.) may exist in vegetable oils and influence the T_1_ and T_2_ relaxation times [41]. The relaxation properties of different vegetable oils can be greatly amplified in the signals acquired by using the pulse sequence in Figure 1. The different relaxation properties were further clearly revealed by the difference in the heat maps in Figure 2.

**Figure 3:**
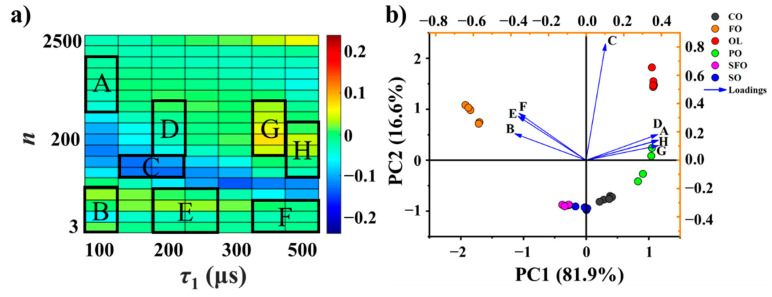
(**a**) The eight regions (A–H) defined in a representative heat map plot for conducting PCA. (**b**) The biplot of the first and second principal components of PCA for 28 vegetable oil samples using the average signal intensity of the eight regions as variables. The ellipses represent the 95% confidence ranges associated with the corresponding edible vegetable oil clusters.

Moreover, the NMR relaxation fingerprints obtained for a given edible oil are very similar and distinctive regardless of the brand involved. The differences present in the fingerprints of the same type of oils but having different brands probably can be attributed to the different production processes and/or the different places of origin of raw material. A study of the influences from the production process and the place of origin is the subject of ongoing studies in our laboratory. Finally, the capture of equivalent *f*(*τ*_1_, *n*) data in the eight regions A–H of the NMR relaxation fingerprint defined in Figure 3a for the same FO sample using the same instrument on the same day but at different times (Figure S5) demonstrates that the proposed method provides highly stable results. We note that based on the experimental parameters described in the experimental section, recording one NMR relaxation fingerprint dataset takes about 15 min totally for one sample.

### 3.2. Characteristic Coordinate System

**Figure 4:**
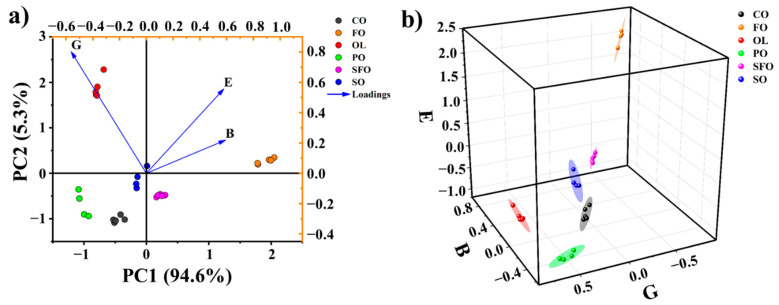
(**a**) The biplot of the first and second principal components of PCA for 28 vegetable oil samples using the three regions B, E, and G as the variables. (**b**) The 3D characteristic coordinate system with six vegetable oil clusters and their corresponding 95% confidence ellipsoids.

The signal intensities of the NMR relaxation fingerprints in the eight regions A–H in Figure 3a were initially used as the feature variables in PCA. These regions were tentatively selected to reduce the amount of data and thus the characteristic dimensions. The selection of these regions was based on visual comparison across the fingerprints of the six oil samples. The application of PCA to the NMR relaxation fingerprints of the 28 vegetable oil samples using the signal intensities of the eight regions as feature variables indicated that the first two principal components (PC1 and PC2) explain 98.5% of the data variance, and therefore represent the original data with reasonable reliability. The biplot results obtained for the 28 different vegetable oil samples using the signal intensities of the eight regions as feature variables are presented in Figure 3b along with the vectors associated with regions A–H in PC1-PC2 space. The biplot presents distinct clusters for each oil type. However, several data points in the SO cluster are very close to those in the SFO cluster, indicating that the two principal components for the eight regions offer insufficient discriminative ability. In order to improve the discriminative ability, the representativeness of the regions was considered. Three regions B, E, and G were focusing solely. The biplot results obtained for the 28 vegetable oils using the signal intensities of the three regions B, E, and G as the feature variables are presented in Figure 4a. It can be observed that the overlap between the SO and SFO clusters has disappeared entirely, and the other four clusters remain reasonably well distinguished from each other. This demonstrates the feasibility and potentiality of the three regions for relaxation fingerprints in differentiating vegetable oil types.

Note that the biplot of PCA using all data in the fingerprints as the variables (i.e., 126 variables from the 18 × 7-matrix) shows similar distinguishing effects for these vegetable oil samples (see Figure S6). This strongly indicates that the selected characteristic regions can be used to well describe the difference in the entire fingerprints of the different oil samples.

The distinctiveness of the clusters associated with the six oil types can be increased considerably by plotting the signal intensities of the 28 NMR relaxation fingerprints in the B, E, and G regions within a 3D coordinate system, as shown in Figure 4b. Also included here are the 95% confidence ellipsoids corresponding to the individual clusters, where the different areas of the ellipsoids can be related to minor variations in the properties of the different oil brands obtained for a given oil type. In this case, even the 95% confidence ellipsoids obtained for the six corresponding vegetable oils are entirely distinct from each other. Accordingly, the 95% confidence ellipsoids can be correlated directly to the different oil types. In this context, it is considered that the confidence ellipsoids can be used for evaluating the genuineness and quality of edible vegetable oils when they are generated from a sufficient number of samples for the same type of oil.

### 3.3. Adulteration Identification

**Figure 5:**
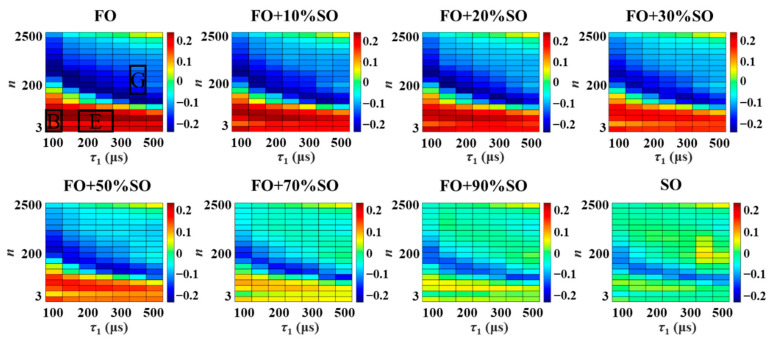
The NMR relaxation fingerprints in the form of heat map plots for FO and SO samples, and a series of mixed FO-SO samples with different adulteration ratios.

**Figure 6:**
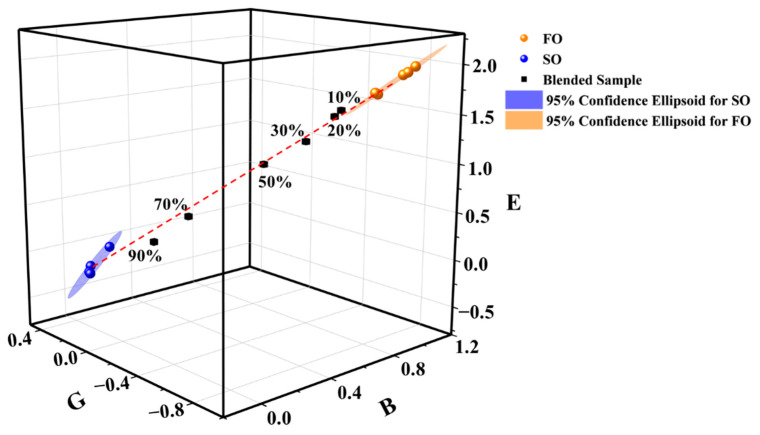
Signal intensities of the B, E, and G regions of the heat map plots in Figure 5 within the 3D characteristic coordinate system with the 95% confidence ellipsoids corresponding to the FO and SO clusters. The red dash line is connected by the center points of the two confidence ellipsoids.

The heat map plots obtained for FO and SO samples, and a series of mixed FO-SO samples with different adulteration ratios are presented in Figure 5. It can be observed that the heat maps vary systematically with an increasing proportion of adulteration. These changes in the heat maps are better represented by their plots within the 3D characteristic coordinate system in Figure 6, along with the 95% confidence ellipsoids corresponding to the FO and SO clusters. Included here is the identifier corresponding to a mixed FO-SO sample with an adulteration ratio of 10%, which is used to demonstrate the limits of the proposed approach.

The adulteration identification can be achieved by monitoring the position of the identifier corresponding to an oil sample in the 3D characteristic coordinate system in Figure 6. This is exemplarily demonstrated by using the identifiers of a series of mixed FO-SO samples. It can be observed from Figure 6 that the identifiers of all adulterated samples with adulteration ratios of 10% or greater reside outside the 95% confidence ellipsoids of FO and SO, and that the identifiers move predictably from the FO cluster to the SO cluster with increasing adulteration ratio. Similar observations were made for the validation dataset samples (Figures S7 and S8). Meanwhile, it can be observed that the mixed samples are distributed near the red dash line connected by the center points of the two confidence ellipsoids, indicating that this approach has possibly potential to identify adulterants in binary mixture oil samples. These results strongly indicate that adulteration in edible vegetable oils can be well-identified using the proposed identifiers and the 95% confidence ellipsoids in the characteristic coordinate system for the mixed FO-SO oils with adulteration ratios of 10% or greater. Moreover, the uniformity in the changes in the identifiers with increasing adulteration ratio suggests that the concentration of the adulterant can be estimated from these results as well.

### 3.4. Quantitative Analysis of Oil Adulteration

The simplest means of conducting a quantitative analysis of adulteration for a binary oil mixture is to assume that the changes in the signal intensities obtained in the B, E, and G regions with respect to changes in the adulteration ratio are linear. Then, univariate regression models can be obtained by fitting the known signal intensities captured for pure and mixed samples in the individual B, E, and G regions to straight lines with respect to the known adulteration ratios. Then, the unknown adulteration ratios of independent samples can be estimated from the regression models based on their captured signal intensities in the individual B, E, and G regions.

**Figure 7:**
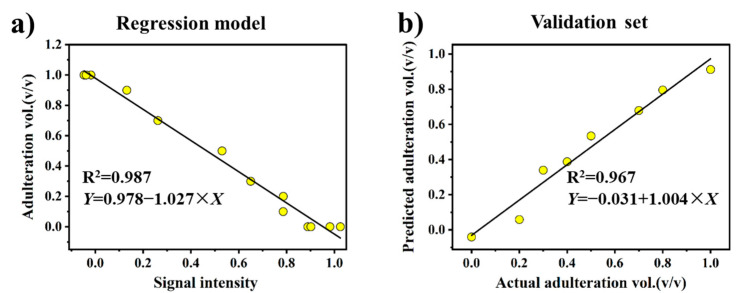
(**a**) The univariate regression model for the signal intensities of FO, SO, and mixed FO-SO samples in region B of the corresponding heat map plots as a function of the adulteration ratio. (**b**) The plot of actual adulteration ratio values versus the values predicted by the univariate regression model.

The individual regression models associated with the B, E, and G regions were established based on the NMR relaxation fingerprints obtained for 3 SO samples, 4 FO samples, and 6 mixed FO-SO samples in the calibration dataset. The remaining 1 SO, 1 FO, and 6 mixed FO-SO samples were used as the validation dataset. The regression model obtained for the adulteration ratio (*Y*) versus the intensity in region B (*X*) is presented in Figure 7a. The regression models obtained from regions E and G are presented in Figure S8. We note that the assumption of linearity is well justified by the coefficient of determination R^2^ value of nearly 1.0. The obtained linear function was applied to establish the *Y* values of the samples in the validation dataset according to their known intensity values *X*, and the predicted values are plotted with respect to their actual values in Figure 7b. A slope of 1.0 for the fitted line would represent a perfect correlation between the actual and predicted values on average. Therefore, a slope value of 1.004 represents an excellent agreement, which is further verified by the corresponding R^2^ value approaching 1.0. The performances of all three regression models are presented in Table 1 according to the root mean square error of calibration (RMSEC) obtained for the calibration dataset and the root mean square error of prediction (RMSEP) obtained for the validation dataset. We note that the low RMSEC and RMSEP values obtained for all three of these models individually represent both good calibration accuracy and good adulteration ratio prediction accuracy, respectively.

**Figure 8:**
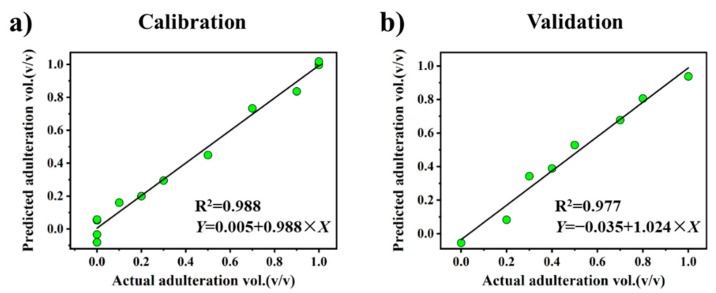
The Plots of the known adulteration values with respect to the predicted values obtained by PLSR models for the different datasets: (**a**) the calibration dataset; (**b**) the validation dataset.

The application of PLSR has been demonstrated to be particularly useful when the predicted variables have severe multicollinearity and the number of samples is less than the number of variables [42]. Accordingly, PLSR was applied in addition to univariate linear regression analysis in this study to establish a regression model with high prediction accuracy (Figure 8). The best PLSR model was obtained with one factor, which has a minimum root mean predicted residual sum of squares (PRESS) [43] value of 0.128 by using cross-validation. The performance of this PLSR model is also listed in Table 1. The corresponding PRESS plot, coefficients plot, and variable importance plot (VIP) are presented in Supporting Information (Figure S8). It is shown that the PLSR model obtained lower RMSEC and RMSEP values than the univariate regression models.

We note that the developed method in the present work still has several limitations in oil adulteration identification. Firstly, the method only provides an approach to rapidly identify the presence of oil adulteration, and is difficult to analyze and identify the possible oil components in an unknown oil sample. In this context, therefore, the method can be considered as a good way for the quality identification of individual types of oils. Secondly, it must be noted that at least one of the components of an oil sample will always be known beforehand, and this should be considered in the analysis. However, this is not a significant issue because oil adulteration is generally restricted to the adulteration of one known specific expensive oil with inexpensive oils. Besides, the method in this work so far was applied only on the binary oil mixtures. In principle, the method should be applicable to the more complicated oil mixtures. The related work is the subject of ongoing studies in our laboratory.

## 4. Conclusions

The present work proposed a simple, accurate, and rapid identification approach for identifying adulteration in edible vegetable oil samples based on their magnetic resonance relaxation fingerprints obtained from LF-NMR spectroscopy measurements. The relaxation fingerprints obtained for six types of edible vegetable oil, including FO, OL, SO, CO, PO, and SFO, were demonstrated by PCA to have sufficiently unique characteristics to enable unambiguous identification. The PCA results were applied for developing a novel 3D coordination system to obtain a unique identifier for the arbitrary vegetable oil samples, which was then demonstrated to provide a direct and simple means of distinguishing between different edible vegetable oil samples when correlated with clusters associated with each specific type of vegetable oil within that characteristic coordination system. It is further demonstrated that the coordination system not only facilitates the identification of individual edible vegetable oil types but can also identify the adulteration of the oil samples comprising two different oils, demonstrating the great potential for the quality identification of individual types of oils. Moreover, the application of univariate regression and partial least squares regression models enables SO concentration in adulterated FO oil samples to be determined easily, accurately, and quantitatively. In future work, we aim to improve the accuracy of this approach by expanding the vegetable oil database with more oil types, more samples within each type, and multiple adulterants for better define clustering in a characteristic coordinate system, thus achieving widespread application of this approach.

## Figures and Tables

**Table 1:** Performance of univariate regression and PLSR (partial least squares regression) models.

		Univariate			PLSR
		Model 1	Model 2	Model 3	
	Input variables	B	E	G	A–H
Accuracy	RMSEP	6.39%	5.88%	5.92%	5.46%
	RMSEC	4.66%	4.66%	6.55%	4.45%
Calibration	Slope	−1.027	−0.515	1.021	0.988
	Intercept	0.978	0.949	0.829	0.005
	R^2^	0.987	0.987	0.974	0.988
Validation	Slope	1.004	0.990	1.027	1.024
	Intercept	−0.031	−0.030	−0.056	−0.035
	R^2^	0.967	0.976	0.984	0.977

## Data Availability

All data needed to evaluate the conclusions in the paper are present in the paper and/or the Supplementary Materials. Additional data related to this paper may be requested from the authors.

## Supplementary Materials

The following are available online at https://www.mdpi.com/article/10.3390/foods10123068/s1, Table S1: T_1_, T_2_ relaxation time of six edible vegetable oils, Figure S1: Relaxation curves of the six edible vegetable oils: (a) T_2_; (b) T_1_, Figure S2: NMR relaxation fingerprints in the form of heat map plots obtained for four different brands of OL and FO, Figure S3: NMR relaxation fingerprints in the form of heat map plots obtained for four different brands of CO and SFO, Figure S4: NMR relaxation fingerprints in the form of heat map plots obtained for three different brands of SO and PO, Figure S5: Results of method stability testing, Figure S6: NMR relaxation fingerprints in the form of heat map plots for FO and SO samples, and a series of mixed FO-SO samples with different adulteration ratios in the validation dataset, Figure S7: Signal intensities of the B, E, and G regions of the heat map plots in Figure S6 within the 3D characteristic coordinate system with the 95% confidence ellipsoids corresponding to the FO and SO clusters, Figure S8: (a) Root mean PRESS values obtained for factors 1–8 from cross validation as a metric for determining the number of significant factors in the PLSR model. (b) Variable importance plot (VIP) of independent variables A–H. (c) Coefficient plot for the PLSR model.
