# Supplementary material for: Rapid Identification of Adulteration in Edible Vegetable Oils Based on Low-Field Nuclear Magnetic Resonance Relaxation Fingerprints
Source: Foods. 2021 Dec 9;10(12):3068. doi: 10.3390/foods10123068 (PMC8701812; doi:10.3390/foods10123068)
Supplement: Supplementary file 1 [file foods-10-03068-s001.zip › foods-1480098-supplementary.pdf]

Supplementary Information

Rapid identification of adulteration in edible vegetable oils based on low-field nuclear magnetic resonance relaxation fingerprints

Zhi-Ming Huang,[1] Jia-Xiang Xin,[1] Shan-Shan Sun,[2] Yi Li,[1] Da-Xiu Wei,[1] Jing Zhu,[1] Xue Lu Wang,[1] Jiachen Wang,[1] Ye-Feng Yao[1*]

[1] Shanghai Key Laboratory of Magnetic Resonance, College of Physics and Electronic Science, East China Normal University, Shanghai 200062, China; 15800959127@163.com (Z.-M.H.); 18366102713@163.com (J.-X.X.); riki941112@gmail.com (Y.L.); dxwei@phy.ecnu.edu.cn (D.-X.W.); jzhu@phy.ecnu.edu.cn (J.Z.); xlwang@phy.ecnu.edu.cn (X.L.W.); jcwang@phy.ecnu.edu.cn (J.W.)

[2] National Institutes for Food and Drug Control, Dongcheng District, Beijing 100050, China; shanshans112@163.com (S.-S.S.)

* Correspondence: yfyao@phy.ecnu.edu.cn

Table S1. T_1 and T_2 relaxation times of six edible vegetable oils.

Edible vegetable oils	T_1 (ms)	T_2 (ms)
Soybean oil (SO)	164.5	150.4
Olive oil (OL)	139.6	119.0
Peanut oil (PO)	145.1	125.5
Flaxseed oil (FO)	193.5	188.7
Corn oil (CO)	158.4	145.0
Sunflower oil (SFO)	176.3	141.3

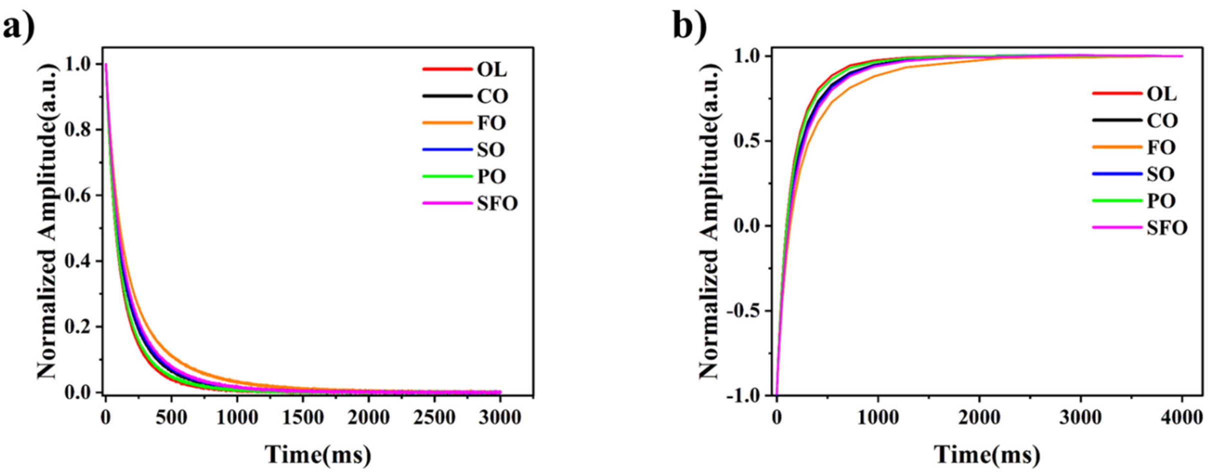

Figure S1. Relaxation curves of the six edible vegetable oils: a) T_2; b) T_1.

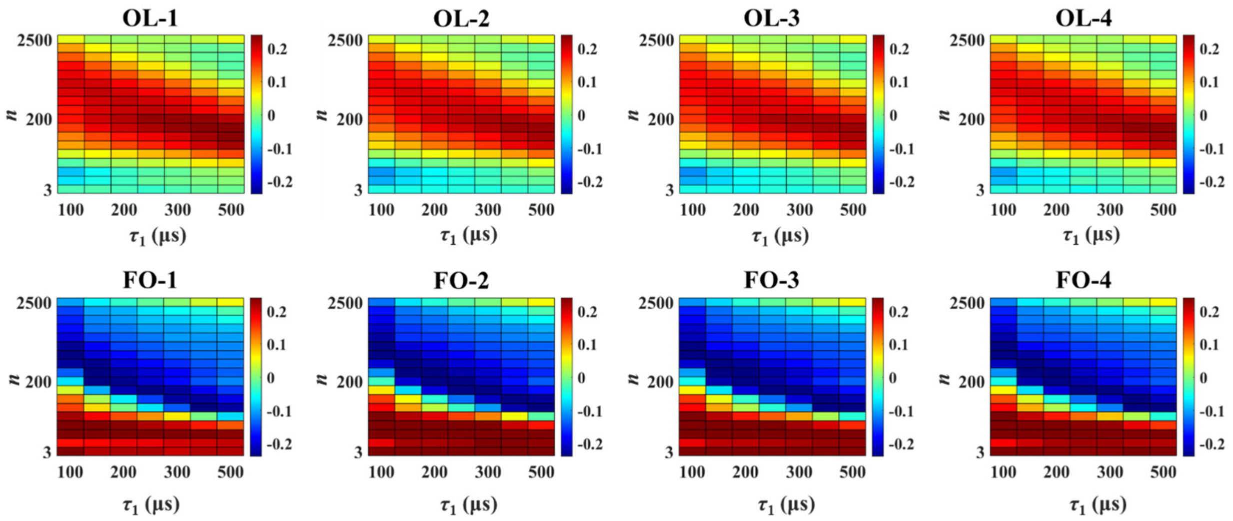

Figure S2. NMR relaxation fingerprints in the form of heat map plots obtained for four different brands of OL and FO.

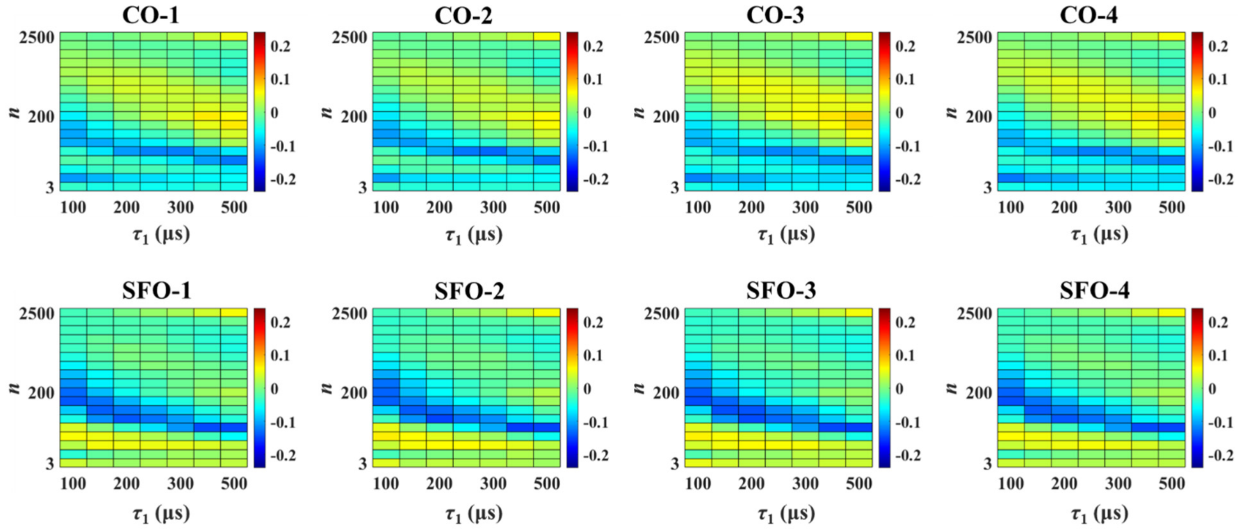

Figure S3. NMR relaxation fingerprints in the form of heat map plots obtained for four different brands of CO and SFO.

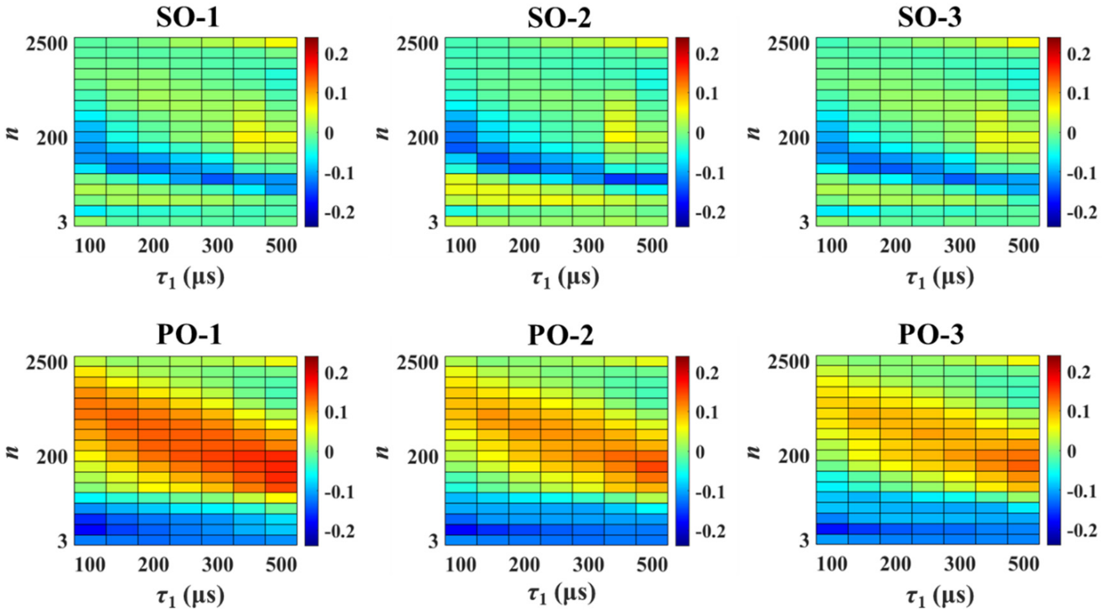

Figure S4. NMR relaxation fingerprints in the form of heat map plots obtained for three different brands of SO and PO.

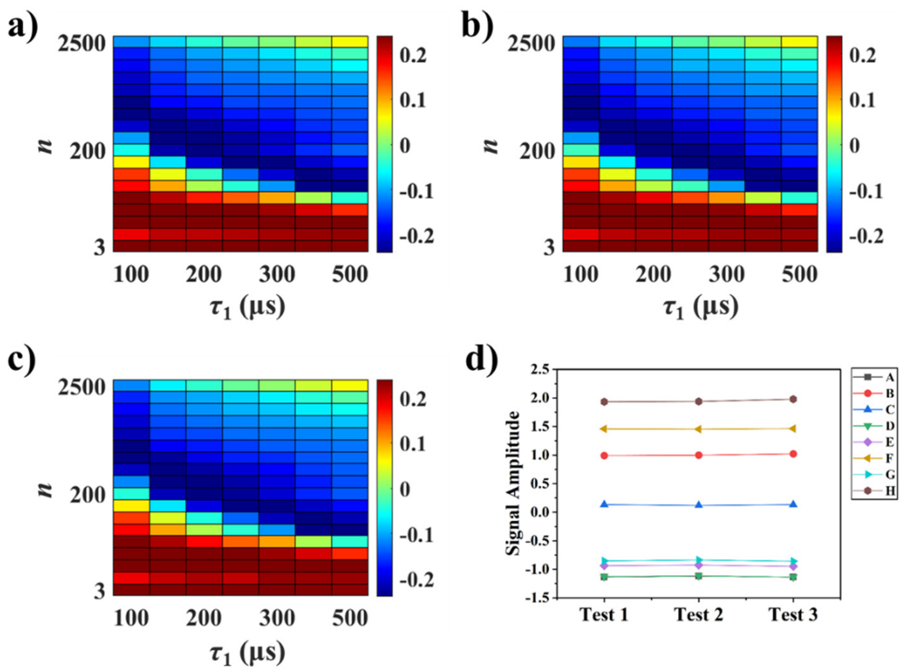

Figure S5. Results of method stability testing, where equivalent $f(\tau_1, n)$ data were acquired for the same FO sample using the same instrument on the same day but at the different times: a) morning (Test 1); b) afternoon (Test 2); c) evening (Test 3). d) Plot of the signal intensity obtained for each of the eight regions defined in Figure 3a for each test.

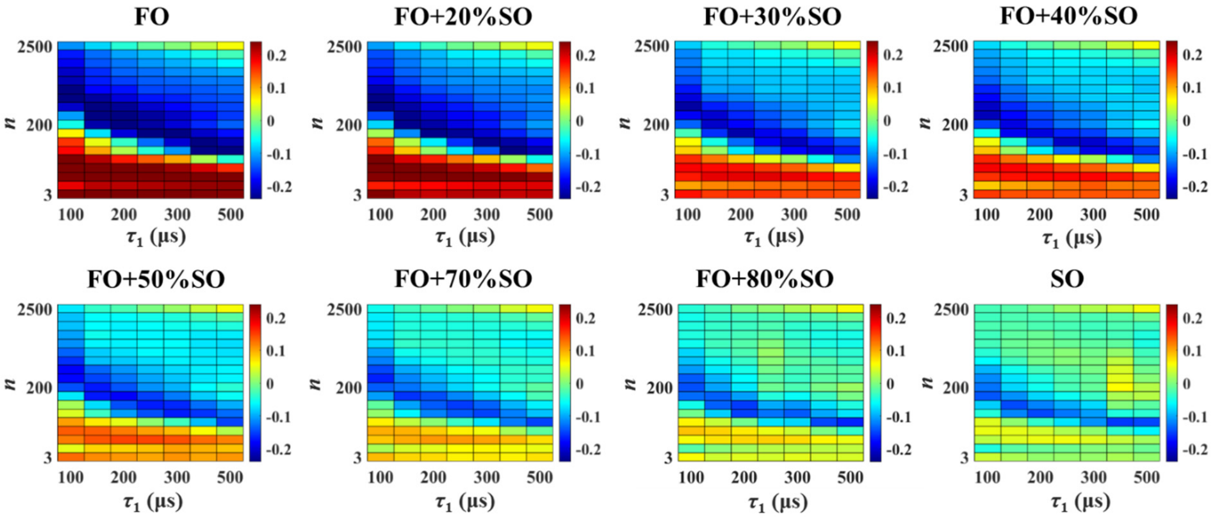

Figure S6. NMR relaxation fingerprints in the form of heat map plots for FO and SO samples, and a series of mixed FO-SO samples with different adulteration ratios in the validation dataset.

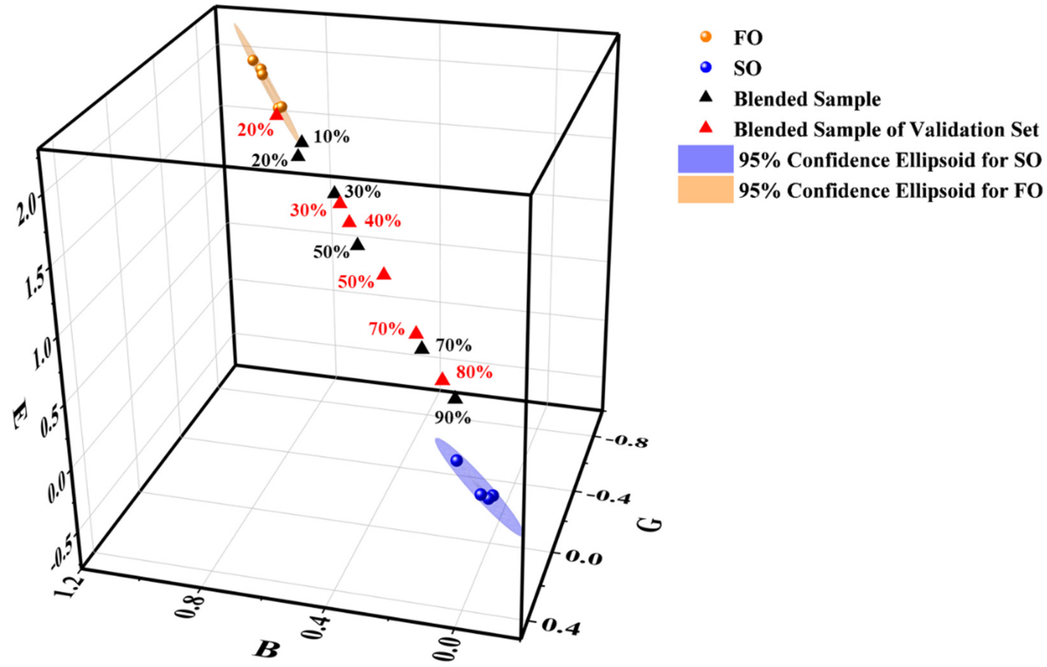

Figure S7. Signal intensities of the B, E, and G regions of the heat map plots in Figure S6 within the 3D characteristic coordinate system with the 95% confidence ellipsoids corresponding to the FO and SO clusters. The data points plotted in Figure 6 are included here for comparison.

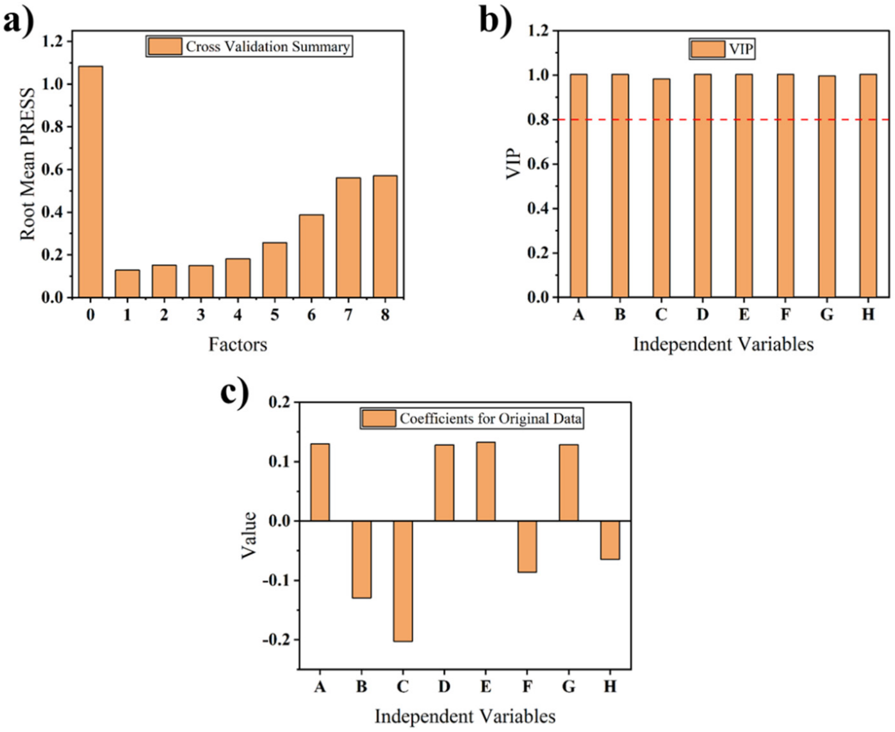

Figure S8. a) Root mean PRESS values obtained for factors 1–8 from cross validation as a metric for determining the number of significant factors in the PLSR model. b) Variable importance plot (VIP) of independent variables A–H. c) Coefficient plot for the PLSR model.
